# Good's buffers have various affinities to gold nanoparticles regulating fluorescent and colorimetric DNA sensing†

**DOI:** 10.1039/d0sc01080d

**Published:** 2020-06-08

**Authors:** Po-Jung Jimmy Huang, Jeffy Yang, Kellie Chong, Qianyi Ma, Miao Li, Fang Zhang, Woohyun J. Moon, Guomei Zhang, Juewen Liu

**Affiliations:** Department of Chemistry, Waterloo Institute for Nanotechnology, University of Waterloo Waterloo ON N2L 3G1 Canada liujw@uwaterloo.ca; School of Chemistry and Chemical Engineering, Shanxi University Taiyuan 030006 China; College of Biological Science and Engineering, Fuzhou University Fuzhou 350108 China

## Abstract

Citrate-capped gold nanoparticles (AuNPs) are highly important for sensing, drug delivery, and materials design. Many of their reactions take place in various buffers such as phosphate and Good's buffers. The effect of buffer on the surface properties of AuNPs is critical, yet this topic has not been systematically explored. Herein, we used halides such as fluoride, chloride, and bromide as probes to measure the relative adsorption strength of six common buffers. Among them, HEPES had the highest adsorption affinity, while MES, citrate and phosphate were weakly adsorbed with an overall ranking of HEPES > PIPES > MOPS > MES > citrate, phosphate. The adsorption strength was reflected from the inhibited adsorption of DNA and from the displacement of pre-adsorbed DNA. This conclusion is also supported by surface enhanced Raman spectroscopy. Furthermore, some buffer molecules did not get adsorbed instantaneously, and the MOPS buffer took up to 1 h to reach equilibrium. Finally, a classic label-free AuNP-based colorimetric sensor was tested. Its sensitivity increased by 15.7-fold when performed in a MES buffer compared to a HEPES buffer. This study has articulated the importance of buffer for AuNP-based studies and how it can improve sensors and yield more reproducible experimental systems.

## Abstract 2

Aside from maintaining pH, Good's buffers can be adsorbed on gold nanoparticles with different affinities, affecting the stability and its fluorescent and colorimetric sensing performance.

## Graphical abstract

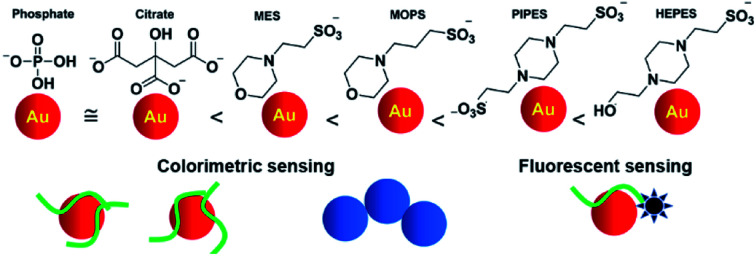

## Introduction

With extremely high extinction coefficients, distance-dependent color, fluorescence quenching, and Raman enhancing properties, gold nanoparticles (AuNPs) have been extensively used in developing optical biosensors.^1–5^ A large fraction of AuNP-based biosensors rely on DNA oligonucleotides such as hybridization probes, aptamers, and DNAzymes for target recognition.^4–9^ In addition, salt is often added to induce the aggregation and color change of AuNPs,^10–12^ facilitate DNA attachment,^13,14^ and promote the hybridization of DNA.^15,16^

An important component of most biochemical reactions as well as biosensors is buffer. The as-prepared AuNPs are often capped by citrate, and the interactions between citrate and AuNPs have been studied in great detail.^17–19^ It is generally thought that citrate is a weak ligand that can be displaced by stronger ligands,^20,21^ although the opposite was also reported to be attributed to the formation of a citrate network on gold.^22^ Other biological buffers, such as the Good's buffers, are often ignored for their interactions with AuNPs as there is little quantitative information available. Recently, Xi and Haes studied the adsorption of HEPES buffer on gold nanostars and found that the protonation of HEPES can weaken its adsorption on gold.^23^ HEPES was studied mainly because it was used to prepare the gold nanostars. Aside from citrate and HEPES, a wide range of buffers have been used for AuNPs, such as phosphate, MES, MOPS, and PIPES. Buffer's effects were also indirectly reflected in the synthesis of AuNPs.^24^

When capped by a strongly adsorbing buffer, AuNPs may have a hard time adsorbing other molecules such as DNA. Many of AuNP-based assays have to rely on DNA adsorption, and such assays include molecular beacons,^25,26^ localized surface plasmon resonance,^27^ SERS,^28–30^ label-free colorimetric assays,^10,11^ catalysis,^31,32^ and even directed growth of nanomaterials.^33–36^ Therefore, the effect of buffer adsorption could be critical. Instead of picking a single buffer for experiments, it is informative to have a quantitative understanding of the adsorption strength of a few common buffers.

However, most buffers do not have a strong optical signature, making it difficult to directly compare their adsorption strength. Fortunately, AuNPs have excellent optical properties that we can use to study the adsorption of various molecules.^37,38^ Halides such as F^−^, Cl^−^, Br^−^, and I^−^ have a gradually increasing interaction strength with gold surfaces.^14,39^ We have used halides to probe the adsorption of nucleosides,^14^ arsenic,^37^ and liposomes on AuNPs.^40^ In this work, we used halides to probe the interaction between buffer molecules and AuNPs. Based on the understanding gained from this study, we have improved the sensitivity of a label-free sensor by 15.7-fold simply by using a different buffer.

## Materials and methods

### Chemicals

All the DNA used in this work were purchased from Integrated DNA Technologies (Coralville, IA, USA) and the sequences are listed in Table S1.† Trisodium citrate dihydrate, citric acid, sodium phosphate monobasic monohydrate, sodium phosphate dibasic heptahydrate, sodium hydroxide, and sodium acetate were obtained from Mandel Scientific (Guelph, ON, Canada). 4-(2-Hydroxyethyl)piperazine-1-ethanesulfonic acid (HEPES), 2-(*N*-morpholino)propanesulfonic acid (MES), 3-(*N*-morpholino)propanesulfonic acid (MOPS), and 1,4-piperazinediethanesulfonic acid (PIPES) were purchased from Bio Basic (Markham, ON, Canada). Gold(iii) chloride, sodium fluoride, sodium chloride, sodium bromide, sodium iodide, hydrochloric acid, and potassium cyanide were from Sigma-Aldrich. The 13 nm and 38 nm AuNPs were synthesized following the well-established citrate reduction protocols.^41,42^ Milli-Q water was used for synthesis and buffer solution preparation.

### Colorimetric titration

The as-synthesized 13 nm AuNPs (particle concentration ∼13 nM) were first diluted with an equal volume of 10 mM of the various buffers and then equilibrated at 23 °C for 1 h before titration. For each sample, 20 μL of sodium halide was quickly mixed with 80 μL of 6.5 nM AuNPs and incubated for 1 min. The UV-vis extinction spectra were then measured using an Agilent 8453 spectrometer. The extinction values at 520 nm and 650 nm were recorded.

### SERS

In a typical experiment, 0.5 mL of the as-synthesized 38 nm AuNPs were mixed with 20 mM various halides (including NaF, NaCl, NaBr, and NaI). After 1 min of incubation, the Raman spectrum was collected using a 785 nm laser excitation with a 10 s integration time (laser power: medium high) on a DeltaNu spectrometer. The nanostars (AuNSs) were synthesized according to the literature.^23^ In brief, 200 μL of 20 μM HAuCl_4_ in Milli-Q water was added to 20 mL of freshly prepared 50 mM HEPES buffer (pH 7.5) and mixed gently for 10 s. The sample was left in the dark at room temperature for approximately 30 min when the solution started turning greenish blue and AuNSs were obtained. They were stored at 4 °C. To remove excess of HEPES buffer, the AuNSs were centrifuged at 14 000 RPM at 15 °C for 30 min to remove the supernatant and replenished by water for a total of four times. For Raman spectroscopy, the AuNSs were added with various concentrations of NaBr and the Raman spectra were acquired after 10 min of incubation.

### DNA adsorption kinetics

The as-synthesized 13 nm AuNPs were first washed to remove the citrate and by-products from the synthesis. Here, the AuNPs were centrifuged at 14 000 RPM for 15 min at 15 °C. The supernatant was removed before re-dispersing them in the same volume of Milli-Q water. After 10 min of incubation at 23 °C, the AuNPs were centrifuged again and the supernatant was replaced by various types of buffers (5 mM, pH 7). We first prepared the buffers at 40 mM and pH 7 as stocks before the total Na^+^ concentration was adjusted to 90 mM by adding NaF. Then, they were each diluted 8-fold to 5 mM to disperse the AuNPs. The exact concentration of the AuNPs was determined *via* UV-vis at 520 nm using the extinction coefficient of 2.7 × 10^8^ M^−1^ cm^−1^. For each sample, 5 nM FAM-labelled DNA was added to 1 nM AuNPs in 5 mM pH 7 buffer that contained 20 mM Na^+^. The sample was excited at 485 nm and the adsorption kinetics were then monitored at 525 nm with a Carey Eclipse fluorescence spectrometer.

### DNA desorption by buffers

For the desorption kinetics study, FAM-T5/AuNP conjugates were prepared using the low-pH method.^43^ 100 μL of the as-synthesized 13 nm AuNPs were added with 2 μL 100 μM FAM-T5 DNA and incubated for about 5 min at room temperature. After that, 2 μL of 500 mM citrate buffer (pH 3) was added and incubated for another 3 min, followed by centrifugation at 14 000 RPM for 10 min and washing 4 times. The obtained precipitate was finally re-suspended in 500 μL of 5 mM phosphate buffer (PB, pH 7). To monitor the kinetics of DNA desorption, 20 μL of the FAM-T5/AuNP conjugates were mixed with 70 μL water and transferred into a cuvette. The background fluorescence was monitored for 2 min (*E*_x_: 485 nm; *E*_m_: 535 nm) before 10 μL of a buffer solution (pH 7.0, 200 mM) was added into the cuvette and the fluorescence was monitored for an additional 38 min, after which 1 μL of KCN (1 M) was added to dissolve the AuNPs and fully release the remaining FAM-T5. For the desorption rate constant calculation, the data were fit with the first-order rate equation *F*_t_ = *F*_0_ + *a*(1 − e^−*kx*^), where *k* is the desorption rate constant. All of the samples were run in duplicate and the standard deviations were calculated accordingly.

### Colorimetric sensing

The AuNPs in the various buffers were prepared following the procedure described above in the adsorption kinetic experiments. Different ratios of DNA probe and target DNA were annealed in buffer (40 mM MES or HEPES with 90 mM Na^+^, pH 7) at 95 °C for 1 min followed by gradual cooling to 23 °C and then storage in ice before usage. For each sample, 100 nM DNA (target concentration can vary) was incubated with 5 nM AuNPs for 30 min. 100 mM NaCl was then added to induce AuNP aggregation. The extinction of the AuNPs at 520 nm and 650 nm were continuously monitored for 35 min with a SpectraMax M3 microplate reader.

## Results and discussion

### Ranking buffer adsorption affinity by halide probes

**Fig. 1:**
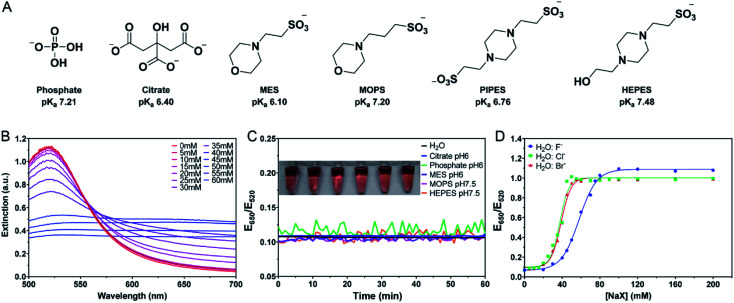
(A) The structures of the buffers used in this work and their first p*K*_a_ values. (B) UV-vis spectra of the as-prepared 13 nm AuNPs diluted 2.5-fold in water and after the addition of various concentrations of NaCl. (C) Kinetics of the color change and a photograph of the AuNPs upon addition of the buffers (4 mM each) measured by the extinction ratio of the AuNPs at 650 nm over 520 nm. (D) Titration curves of the water diluted AuNPs with three different halide salts.

The AuNPs used in this work were prepared by the classic citrate reduction method (1 mM HAuCl_4_ + 10% volume of 38.8 mM trisodium citrate).^41,44^ Since each equivalent of citrate can reduce ∼4 equivalents of HAuCl_4_,^45^ ∼3.3 mM citrate remained in the as-synthesized 13 nm AuNPs. The AuNPs were stabilized by the adsorption of negatively charged citrate and appear red. Upon addition of salt, the charge is screened and the AuNPs can approach each other. Once the short-ranged attractive van der Waals forces start to dominate, the AuNPs would aggregate and change their color to blue or purple. The UV-vis spectra of the AuNPs with increasing concentrations of NaCl are shown in Fig. 1B, where the 520 nm plasmon peak gradually decreased and the absorption in the longer wavelength region increased. We can use the extinction ratio at 650 nm over that at 520 nm to quantify the color of the sample.

Most AuNP-related reactions are carried out in phosphate buffers (*e.g.* PBS) or various Good's buffers, and adsorption of these buffers on AuNPs may affect their colloidal stability. The buffers studied in this work are shown in Fig. 1A. Some buffer molecules contain nitrogen atoms with a lone pair of electrons that may get adsorbed on AuNPs.^23^ A main goal of this work is to rank the adsorption affinity of these buffers and to understand the effect of buffer on DNA adsorption. By adding a final 5 mM of buffer, the AuNPs remained stable for 1 h in all these buffers (Fig. 1C). Although tris(hydroxymethyl)aminomethane (Tris) is also a common buffer, we excluded it from this study since Tris caused aggregation of AuNPs and thus is not applicable for studying bare AuNPs (Fig. S1†).

If a buffer can stabilize AuNPs, the required salt concentration to induce its aggregation or color change would be higher. We quantitatively compared the color change as a function of salt type and concentration. For example, we plotted the extinction ratio from Fig. 1B in Fig. 1D (green squares), and a sigmoidal response was obtained. The AuNPs were stable when the NaCl concentration was below 20 mM, and the color quickly changed between 20 and 50 mM, after which the effect of the salt was saturated. By fitting the data to a sigmoidal equation, we obtained the midpoint of the transition and defined it as AC_50_ (37.6 mM NaCl). A similar result was obtained for NaBr (38.9 mM), while NaF had a higher AC_50_ of 57.8 mM (Fig. 1D). Since these salts all had the same Na^+^ cation, the only difference was the anions. We explained this trend based on Cl^−^ and Br^−^ displacing the surface citrate. After the displacement, Cl^−^ and Br^−^ allowed the AuNPs to approach closer to each other (halides are smaller than citrates). F^−^ cannot displace the citrate, and thus a higher NaF concentration was needed. The implication is that citrate adsorption might be weaker than that of Cl^−^ and Br^−^, but stronger than F^−^. By looking at the pattern of the color change, we may use the halides to rank the adsorption affinity of the buffers.

**Fig. 2:**
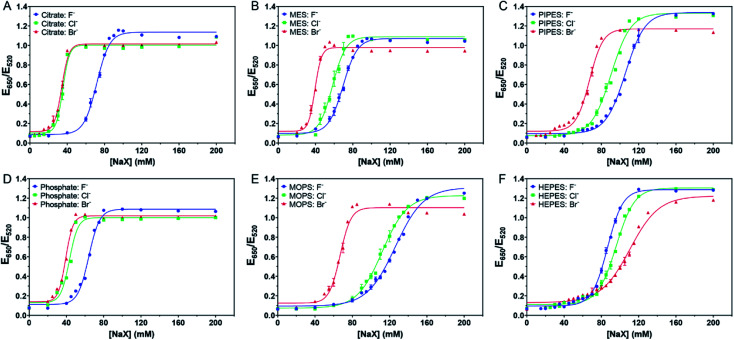
Titration curve of 5.2 nM AuNPs in 4 mM of (A) citrate with pH 6, (B) MES with pH 6, (C) PIPES with pH 7, (D) phosphate with pH 6, (E) MOPS with pH 7.5, and (F) HEPES with pH 7.5 buffers.

In this sample, the concentration of citrate was only ∼1.3 mM since the original AuNPs with ∼3.3 mM of citrate were diluted by 2.5-fold for this study. We then added more citrate (total 4 mM citrate, Fig. 2A). The pattern still looked the same. While it required similar concentrations of NaCl (AC_50_ = 35.3 mM) or NaBr (AC_50_ = 34.0 mM) to induce aggregation, the required NaF increased (AC_50_ = 71.7 mM). The extra citrate could not overcome displacement by Cl^−^ or Br^−^, but was effective against F^−^. Therefore, we can conclude that the adsorption affinity of citrate is stronger than that of F^−^.

We then tested the four Good's buffers plus phosphate, and they all showed different patterns of responses to the halides. MES (Fig. 2B) and PIPES (Fig. 2C) displayed an equal spacing for the three halides, suggesting that their affinities to the AuNPs might be close to that of Cl^−^. In other words, these two buffers might be adsorbed more strongly than citrate on the AuNPs. The behavior of phosphate (Fig. 2D) was similar to that of citrate, both weaker than Cl^−^. For MOPS (Fig. 2E), the response to Cl^−^ was very close to that of Br^−^, suggesting that MOPS might be adsorbed more strongly than Cl^−^ (certainly stronger than MES and PIPES), although still weaker than Br^−^.

Very interestingly, HEPES showed an opposite trend (Fig. 2F), with the highest stability observed with Br^−^. At 80 mM of NaBr, the AuNPs remained stable, and thus Br^−^ provided extra stability. We suspected that Br^−^ cannot fully displace HEPES and that Br^−^ and HEPES could be co-adsorbed on the surface. We previously observed that Br^−^ could be co-adsorbed with DNA.^14^ Therefore, HEPES appeared to have the strongest affinity among the tested buffers.

### A quantitative ranking index

**Fig. 3:**
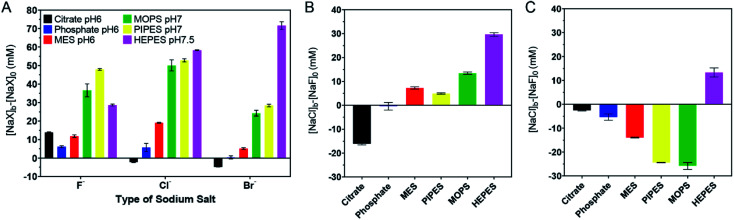
Quantitative ranking of buffer adsorption affinity. (A) Comparison of the extra salt needed to aggregate the 4 mM buffer protected AuNPs. (B) Comparison of NaCl and NaF for aggregating the AuNPs in each buffer. (C) Comparison of NaBr and NaCl for aggregating the AuNPs in each buffer. The original data are in Fig. 2.

The above work can roughly estimate the relative adsorption strength of the buffers. We also wanted to quantitatively rank them. We took the response to the as-prepared AuNPs shown in Fig. 1D as the reference points and defined its AC_50_ values as [NaF]_0_, [NaCl]_0_, and [NaBr]_0_, respectively. For the samples with buffers added, we then defined their AC_50_ values as [NaF]_*b*_, [NaCl]_*b*_, and [NaBr]_*b*_. If the newly added buffer had a stabilizing effect, the difference should be positive (*e.g.* [NaF]_*b*_ − [NaF]_0_). The larger the difference, the higher the adsorption stability. We plotted the results in Fig. 3A, and a few observations were made. First, most bars in Fig. 3A are positive, indicating that the extra buffers stabilized the AuNPs. Second, the bars for citrate, phosphate, and MES are shorter than those for MOPS, PIPES, and HEPES, indicating that the latter three buffers were more strongly adsorbed.

We then compared the height of the bars of the neighboring buffers. By comparing the Cl^−^ and F^−^ data (Fig. 3B), citrate needed more F^−^, HEPES and MOPS needed a lot more Cl^−^ (>10 mM), while the rest needed slightly more Cl^−^ (∼5 mM or less) to reach the AC_50_. The need for more Cl^−^ can be interpreted as the buffer being adsorbed more stably than Cl^−^, and Cl^−^ acted as a stabilizer instead of a competitor. When Cl^−^ and Br^−^ were compared (Fig. 3C), only the HEPES bar was positive, indicating its stronger adsorption compared to Br^−^. Based on these quantitative measurements, we ranked the adsorption stability to be HEPES > MOPS > PIPES > MES > citrate > phosphate.

The ranking above was from the colloidal stability of the AuNPs, which did not directly reflect the intrinsic thermodynamic stability. This study highlighted the importance of including all three halide salts. For example, by just looking at NaF, PIPES might appear to be more strongly adsorbed than HEPES. Since F^−^ cannot displace either of them, this difference reflected the protection effect of the buffers (*i.e.* PIPES is a better stabilizer). Despite the fact that PIPES had a better protection effect, in the presence of NaBr, the AuNPs were much more resistant to aggregation in HEPES. Combining these two, we can conclude that HEPES was adsorbed more strongly.

From a structural standpoint, HEPES has two nitrogen atoms each with a lone pair electrons that may coordinate with the AuNP surface.^23^ MES is a more weakly adsorbed buffer and it has only one nitrogen atom. HEPES and PIPES are very similar, except that the hydroxyl group in HEPES is replaced by a sulfonate in PIPES. The negatively charged sulfonate might exert charge repulsion on the AuNPs, causing it to be less stably adsorbed.

While cations in salts were believed to be the main factor for inducing AuNP aggregation *via* charge screening, anions can be either protective by co-adsorption with buffers or destabilizing by displacing weakly adsorbed buffers. Practically, we need to consider both the cation and anion part of the salts.

The above work was all performed at pH 6 or 7, close to their respective p*K*_a_'s. We then picked two buffers and varied the pH (Fig. S2†). In general, the higher the pH, the more salt was required. This can be understood as by increasing the pH, the surface became more negatively charged and thus more salt was needed to induce aggregation. The trends for the three halide salts remained the same regardless of pH.

### Buffer adsorption studied by Raman spectroscopy

**Fig. 4:**
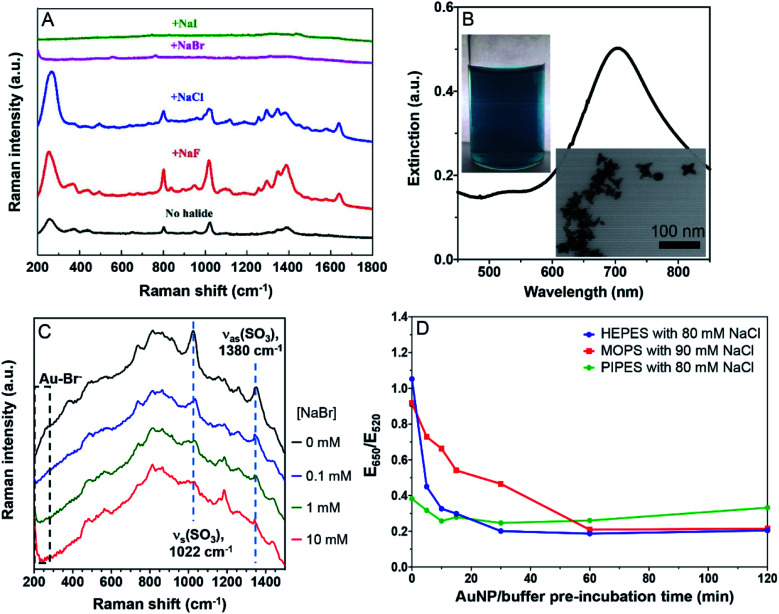
(A) SERS spectra of citrate-capped AuNPs in the presence of 20 mM of the various halides. (B) The UV-vis spectrum of the AuNSs, their photograph and a TEM micrograph. (C) SERS spectra of the HEPES-capped AuNSs with various concentrations of Br^−^. The Br^−^ peak is below 200 cm^−1^ and only its tail showed up. The two highlighted SO_3_ peaks are from HEPES. (D) Effect of pre-incubation time of AuNP/buffer reflecting the adsorption kinetics of the buffers. The 13 nm AuNPs were incubated with a buffer first for designated times before reacting with NaCl for 1 min and UV-vis measurement.

To further study the displacement of the buffers by halides, we then performed Raman spectroscopy, taking advantage of the surface enhanced Raman scatter (SERS) effect of AuNPs. The as-prepared AuNPs had a few weak Raman peaks (Fig. 4A, black spectrum). The broad peak at 250 cm^−1^ was a signature of Cl^−^ adsorption on the AuNPs, while the other peaks were assigned to the adsorbed citrate. Adding 20 mM NaF increased the intensity of all the peaks, attributable to the aggregation of the AuNPs. Adding NaCl increased the Cl^−^ peak but weakened the others, suggesting the displacement of a fraction of citrate by Cl^−^. Adding Br^−^ and I^−^ fully silenced the spectra, suggesting these two halides fully displaced the adsorbed Cl^−^ and citrate. Thus Br^−^ was adsorbed more strongly than citrate, consistent with the above colorimetric titration data.

We then tested the strongly adsorbing HEPES. Since the SERS signal of HEPES was too weak on the AuNPs, we synthesized gold nanostars (AuNSs).^23^ Their green color, a 700 nm surface plasmon peak, and the TEM micrograph all indicated the successful preparation (Fig. 4B). To boost the Raman signal, the AuNSs were washed to remove the excess HEPES (Fig. S3†).^23^ With increasing concentration of NaBr, the peak beyond 200 cm^−1^ grew significantly due to the Au–Br bond (limited by the range of our instrument, we could only see the tail part of the peak). Even with 10 mM Br^−^, the characteristic peaks of HEPES remained, suggesting that HEPES and Br^−^ were co-adsorbed on the AuNSs (Fig. 4C). This is also consistent with our colorimetric titration results.

**Fig. 5:**
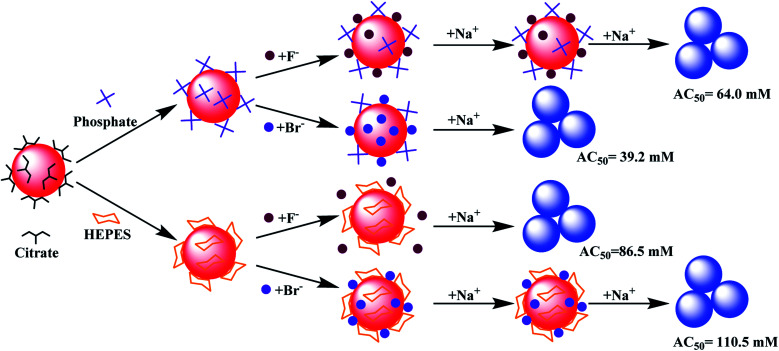
(Top) A model showing the displacement of adsorbed weak buffers such as phosphate and then aggregation caused by Na^+^, or the buffer is not displaced (*e.g.* by F^−^) followed by aggregation at a higher concentration of Na^+^. (Bottom) Strongly adsorbed HEPES is not displaced by F^−^ or Br^−^, and the co-adsorbed Br^−^ further stabilized the AuNPs.

Based on the above results, models of interactions are shown in Fig. 5 using two extreme cases. For buffers weaker or similar to citrate, the AuNPs would aggregate by adding ∼40 mM NaBr (AC_50_ value). Citrate displacement by Br^−^ is an important aspect of the reaction since ∼60 mM NaF was needed (Fig. 5, top). When capped by a stronger buffer (*e.g.* HEPES), a higher salt concentration was required to aggregate the AuNPs. The needed salt concentration is even higher if the halide part of the salt cannot displace the buffer, but can be co-adsorbed with the buffer (*e.g.* HEPES and Br^−^, Fig. 5, bottom).

### Kinetics of buffer adsorption

The above experiments were performed after a prolonged incubation of the AuNPs with the buffers. The buffer molecules are small and thus they are expected to be rapidly adsorbed on the AuNPs, enabling the system to reach equilibrium quickly. Indeed, PIPES did not show time-dependent effects (Fig. 4D, green trace). However, it took about 30 min for the HEPES sample to reach equilibrium, while about 1 h was needed for MOPS. Therefore, to obtain consistent results, the incubation time needs to be sufficiently long for some buffers.

HEPES is known to be adsorbed at different conformations on the AuNP surface. Based on our data, we deduced that the conformational changes may explain the slow adsorption kinetics of HEPES and MOPS. Therefore, this time scale of up to 1 h reflected buffer adsorption. It was recently reported that AuNPs can also help oxidize HEPES, thus producing a reactive oxygen species, but this reaction is at an even longer time scale, taking days to observe.^46^

### Effect of buffers on fluorescent DNA sensing

**Fig. 6:**
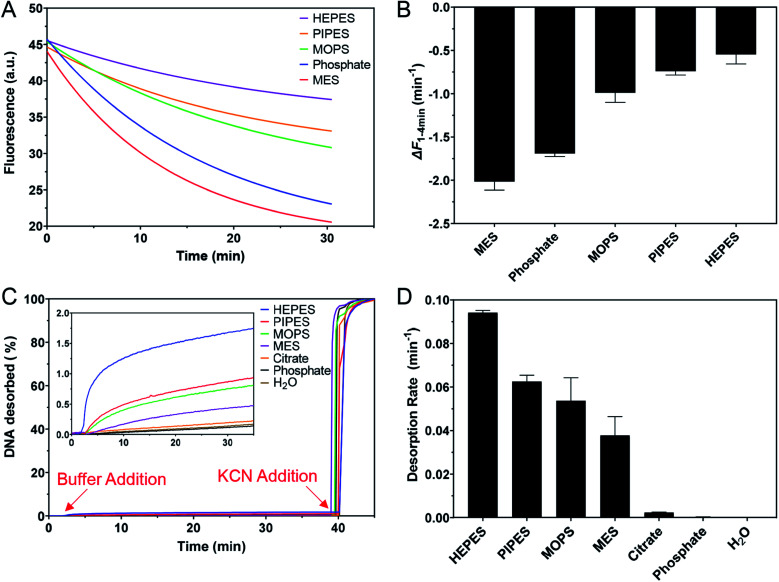
(A) Adsorption kinetics of 5 nM FAM-A_15_ DNA onto 1 nM AuNPs in 5 mM pH 7 buffer containing 20 mM Na^+^ (adjusted by adding NaF). (B) The initial rate of fluorescence quenching (1–4 min). (C) The kinetics of FAM-T_5_ DNA desorption from the AuNPs in the presence of various buffers at pH 7.0 with 20 mM Na^+^. Inset: the increase in fluorescence after adding the buffers with a small scale y-axis. (D) Rate constants of DNA desorption by these buffers.

Buffer adsorption can change the surface properties of AuNPs, which would consequently have a profound impact on their applications. Here, we studied the adsorption of DNA oligonucleotides as an example. When FAM-labeled A_15_ DNA was incubated with the AuNPs in various buffers, we found very different rates of DNA adsorption revealed by its fluorescence quenching (Fig. 6A). The slowest adsorption occurred in HEPES, while the fastest occurred in MES (Fig. 6B), consistent with the ranking of buffer adsorption affinity. Since DNA adsorption relies on the direct interaction between DNA bases and the gold surface,^13,47–49^ the buffer layer needs to be displaced by DNA, and strongly adsorbed buffers are more difficult to displace.

To further study the affinity of buffer adsorption, we made FAM-T_5_ to be pre-adsorbed on the AuNPs. We chose T_5_ DNA (5 thymines) since it has a weak affinity and can be more easily displaced.^26,47,50^ The kinetics of fluorescence enhancement after buffer addition (at 2 min) were then monitored (Fig. 6C). After another 38 min, KCN was added to dissolve the AuNPs and fully release the remaining FAM-T_5_. Since the overall desorption was quite small (<2%), the section where buffer displacement occurred is shown in the inset of Fig. 6C with the order of HEPES > PIPES > MOPS > MES > citrate, phosphate. To quantitatively exhibit the difference between buffers, the desorption rate was calculated (Fig. 6D). Since the overall desorbed DNA was very low (<2%), it is likely that only the relatively weakly adsorbed DNA strands were displaced.^26^ Taking the data in Fig. 6 together, we can conclude that the buffer molecules have stronger kinetics effects on DNA adsorption, while thermodynamically, DNA adsorption is still much stronger (DNA can displace the buffers to be adsorbed, but the reverse reaction is more difficult).

### Weaker buffers for more sensitive colorimetric sensors

**Fig. 7:**
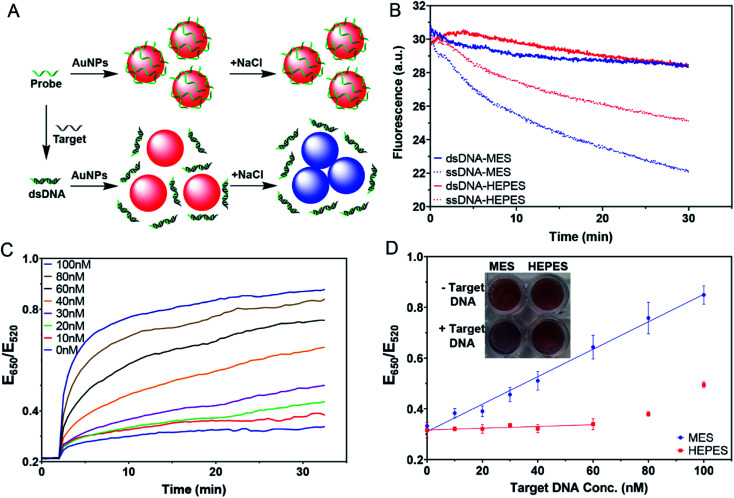
(A) A scheme illustrating colorimetric detection of DNA. (B) The kinetics of FAM-24-mer DNA and its duplex adsorption in MES and HEPES, pH 7. (C) Kinetics of color change of the sensing samples in the presence of various concentrations of the target DNA in MES buffer, pH 7. (D) Calibration curves of the detection in MES and HEPES buffers measured at 20 min after adding NaCl. Inset: a photograph of 4 nM AuNPs and 100 nM probe DNA with 0 nM or 60 nM target DNA in 5 mM MES or HEPES buffer after 20 min incubation with 100 mM NaCl, pH 7.

One of the most important applications of DNA adsorption by AuNPs is the label-free colorimetric detection of DNA. The general sensing scheme is shown in Fig. 7A, where the free single-stranded DNA (ssDNA) probe can quickly be adsorbed on the AuNPs and protect the AuNPs from salt-induced aggregation. On the other hand, with the complementary target DNA, the probe would hybridize to form a double-stranded DNA (dsDNA); however, the adsorption of the dsDNA is slow. As a result, the AuNPs would aggregate upon salt addition, yielding a blue color. This method has been extensively used for the detection of complementary nucleic acids and later for other targets using aptamer probes.^9,10,45–47^ However, the effect of buffer was not systematically understood.

We first compared the adsorption kinetics of single and double-stranded DNA in different buffers. A FAM-labeled 24-mer DNA was used either alone or pre-hybridized with its cDNA. We chose MES and HEPES to represent the weak and strong buffers, respectively. In both cases, the adsorption of the single-stranded probe was faster than the adsorption of the duplex (Fig. 7B), consistent with the model shown in Fig. 7A. The difference was greater in MES, and thus MES should be a better buffer for this sensing application.

We then tested the colorimetric sensor for the detection of target DNA using AuNPs as shown in Fig. 7A. To demonstrate the feasibility of this design, samples with different molar ratios of complementary DNA strands were pre-annealed and then incubated with AuNPs for 30 min. 100 mM NaCl was then added to induce AuNP aggregation. The kinetics of the color change for the MES buffer were then plotted (Fig. 7C), showing that the more the target was added, the faster the color changed to blue. In the inset of Fig. 7D, we can see that MES showed different colors, while HEPES did not.

With 100 nM probes, the sensor in MES buffer showed a linear dependence from 0–100 nM by plotting the ratio at 20 min (Fig. 7D). The sensor had a limit of detection of 3.4 nM based on the 3*σ*/slope, where *σ* is the standard deviation of the background variation. On the other hand, we barely observed any response in the HEPES buffer when the target concentration was below 60 nM. This suggested that even the remaining probe DNA could act together with adsorbed HEPES to stabilize the AuNPs. When the target concentration was too high, the remaining free DNA was too low, and HEPES alone was insufficient to protect the AuNPs. This can explain the increased ratio for the HEPES sample after 60 nM target DNA. Therefore, for this type of sensing experiment, using the weakly adsorbing MES buffer is recommended, while the strongly adsorbing HEPES buffer needs to be avoided to obtain higher sensitivity.

## Conclusions

In summary, we ranked the adsorption affinity of a few common buffers on AuNPs using several halides as probes. This assay took advantage of the colloidal stability, color, and different halide interaction strengths of the AuNPs. Among them, HEPES had the highest adsorption affinity, while MES and phosphate were weakly adsorbed. The adsorption affinity ranking was also confirmed using DNA probes by both inhibiting the adsorption of DNA, and by displacing pre-adsorbed DNA. The weakly adsorbed buffers appeared also to take a longer time to reach equilibrium. With this understanding, we showed that the sensitivity of a classic label-free colorimetric sensor can increase by nearly 16-fold simply by changing the buffer from HEPES to MES. This study is important since it articulates the critical role of buffers, which was neglected in most previous AuNP-related analytical and materials studies. The careful control of buffers can ensure more reproducible results and better AuNP-based hybrid materials.

## Conflicts of interest

There are no conflicts to declare.

## Supplementary Material

SC-011-D0SC01080D-s001

## References

[cit1] Rosi N. L., Mirkin C. A. (2005). Chem. Rev..

[cit2] Saha K., Agasti S. S., Kim C., Li X., Rotello V. M. (2012). Chem. Rev..

[cit3] Aldewachi H., Chalati T., Woodroofe M. N., Bricklebank N., Sharrack B., Gardiner P. (2018). Nanoscale.

[cit4] Liu J., Cao Z., Lu Y. (2009). Chem. Rev..

[cit5] Liu B., Liu J. (2019). Matter.

[cit6] Zhao W., Brook M. A., Li Y. (2008). ChemBioChem.

[cit7] Song S. P., Qin Y., He Y., Huang Q., Fan C. H., Chen H. Y. (2010). Chem. Soc. Rev..

[cit8] Zhang J. N., Liu B., Liu H. X., Zhang X. B., Tan W. H. (2013). Nanomedicine.

[cit9] Ma L., Liu J. (2020). iScience.

[cit10] Li H., Rothberg L. J. (2004). J. Am. Chem. Soc..

[cit11] Li H., Rothberg L. (2004). Proc. Natl. Acad. Sci. U. S. A..

[cit12] Liu B., Kelly E. Y., Liu J. (2014). Langmuir.

[cit13] Zhang X., Servos M. R., Liu J. (2012). Langmuir.

[cit14] Liu B., Wu P., Huang Z., Ma L., Liu J. (2018). J. Am. Chem. Soc..

[cit15] Jin R., Wu G., Li Z., Mirkin C. A., Schatz G. C. (2003). J. Am. Chem. Soc..

[cit16] Macfarlane R. J., Thaner R. V., Brown K. A., Zhang J., Lee B., Nguyen S. T., Mirkin C. A. (2014). Proc. Natl. Acad. Sci. U. S. A..

[cit17] Park J.-W., Shumaker-Parry J. S. (2014). J. Am. Chem. Soc..

[cit18] Al-Johani H., Abou-Hamad E., Jedidi A., Widdifield C. M., Viger-Gravel J., Sangaru S. S., Gajan D., Anjum D. H., Ould-Chikh S., Hedhili M. N., Gurinov A., Kelly M. J., El Eter M., Cavallo L., Emsley L., Basset J.-M. (2017). Nat. Chem..

[cit19] Spina R. L., Spampinato V., Gilliland D., Ojea-Jimenez I., Ceccone G. (2017). Biointerphases.

[cit20] Perera G. S., Athukorale S. A., Perez F., Pittman C. U., Zhang D. (2018). J. Colloid Interface Sci..

[cit21] Rani M., Moudgil L., Singh B., Kaushal A., Mittal A., Saini G. S. S., Tripathi S. K., Singh G., Kaura A. (2016). RSC Adv..

[cit22] Park J.-W., Shumaker-Parry J. S. (2015). ACS Nano.

[cit23] Xi W., Haes A. J. (2019). J. Am. Chem. Soc..

[cit24] Ahmed S. R., Oh S., Baba R., Zhou H., Hwang S., Lee J., Park E. Y. (2016). Nanoscale Res. Lett..

[cit25] Maxwell D. J., Taylor J. R., Nie S. (2002). J. Am. Chem. Soc..

[cit26] Zhang F., Wang S., Liu J. (2019). Anal. Chem..

[cit27] Bolduc O. R., Masson J. F. (2011). Anal. Chem..

[cit28] Scarabelli L., Coronado-Puchau M., Giner-Casares J. J., Langer J., Liz-Marzan L. M. (2014). ACS Nano.

[cit29] Gearheart L. A., Ploehn H. J., Murphy C. J. (2001). J. Phys. Chem. B.

[cit30] Jamieson L. E., Asiala S. M., Gracie K., Faulds K. and Graham D., in Annu. Rev. Anal. Chem., ed. R. G. Cooks and J. E. Pemberton, 2017, vol. 10, pp. 415–437 10.1146/annurev-anchem-071015-041557 28301754

[cit31] Wang Y. L., Fang L. L., Gong M., Deng Z. X. (2019). Chem. Sci..

[cit32] Hizir M. S., Top M., Balcioglu M., Rana M., Robertson N. M., Shen F. S., Sheng J., Yigit M. V. (2016). Anal. Chem..

[cit33] Satyavolu N. S. R., Tan L. H., Lu Y. (2016). J. Am. Chem. Soc..

[cit34] Tan L. H., Yue Y., Satyavolu N. S. R., Ali A. S., Wang Z., Wu Y., Lu Y. (2015). J. Am. Chem. Soc..

[cit35] Shen J. L., Xu L. F., Wang C. P., Pei H., Tai R. Z., Song S. P., Huang Q., Fan C. H., Chen G. (2014). Angew. Chem., Int. Ed..

[cit36] Yao G. B., Li J., Li Q., Chen X. L., Liu X. G., Wang F., Qu Z. B., Ge Z. L., Narayanan R. P., Williams D., Pei H., Zuo X. L., Wang L. H., Yan H., Feringa B., Fan C. H. (2019). Nat. Mater..

[cit37] Zong C., Zhang Z., Liu B., Liu J. (2019). Langmuir.

[cit38] Wang F., Curry D. E., Liu J. (2015). Langmuir.

[cit39] Meena S. K., Celiksoy S., Schafer P., Henkel A., Sonnichsen C., Sulpizi M. (2016). Phys. Chem. Chem. Phys..

[cit40] Liu X., Li X., Xu W., Zhang X., Huang Z., Wang F., Liu J. (2018). Langmuir.

[cit41] Liu J., Lu Y. (2006). Nat. Protoc..

[cit42] Frens G. (1973). Nature.

[cit43] Zhang X., Servos M. R., Liu J. (2012). J. Am. Chem. Soc..

[cit44] Storhoff J. J., Elghanian R., Mucic R. C., Mirkin C. A., Letsinger R. L. (1998). J. Am. Chem. Soc..

[cit45] Leng W., Pati P., Vikesland P. J. (2015). Environ. Sci.: Nano.

[cit46] Wang L., Wan Y., Xu Q., Lou X. (2019). Langmuir.

[cit47] Kimura-Suda H., Petrovykh D. Y., Tarlov M. J., Whitman L. J. (2003). J. Am. Chem. Soc..

[cit48] Herne T. M., Tarlov M. J. (1997). J. Am. Chem. Soc..

[cit49] Pei H., Li F., Wan Y., Wei M., Liu H., Su Y., Chen N., Huang Q., Fan C. (2012). J. Am. Chem. Soc..

[cit50] Storhoff J. J., Elghanian R., Mirkin C. A., Letsinger R. L. (2002). Langmuir.

