# Supplementary material for: Good's buffers have various affinities to gold nanoparticles regulating fluorescent and colorimetric DNA sensing
Source: Chem Sci. 2020 Jun 8;11(26):6795–804. doi: 10.1039/d0sc01080d (PMC8159396; doi:10.1039/d0sc01080d)
Supplement: SC-011-D0SC01080D-s001 [file SC-011-D0SC01080D-s001.pdf]

Electronic Supplementary Material (ESI) for Chemical Science.
This journal is © The Royal Society of Chemistry 2020

Electronic Supplementary Information

Good's buffers have various affinities on gold nanoparticles regulating fluorescent and colorimetric DNA sensing

Po-Jung Jimmy Huang[1], Jeffy Yang[1], Kellie Chong[1], Qianyi Ma[1], Miao Li[1,2], Fang Zhang[1,3], Woohyun J. Moon[1], Guomei Zhang[2] and Juewen Liu[1]*

1. Department of Chemistry, Waterloo Institute for Nanotechnology, University of Waterloo, Waterloo, ON, N2L 3G1, Canada

Email: liujw@uwaterloo.ca

2. School of Chemistry and Chemical Engineering, Shanxi University, Taiyuan, 030006 China

3. College of Biological Science and Engineering, Fuzhou University, Fuzhou, 350108, China

Table S1. List of the DNA sequences used in this work. /56-FAM/ means carboxyfluorescein.

**DNA Name**	**Sequence** (From 5' to 3')
FAM-A15	/56-FAM/AAAAAAAAAAAAAAA
FAM-T15	/56-FAM/TTTTTTTTTTTTTTT
FAM-T5	/56-FAM/TTTTT
FAM-24mer	/56-FAM/ACGCATCTGTGAAGAGAAACTGGG
24mer	ACGCATCTGTGAAGAGAAACTGGG
24mer-cDNA	CCCAGGTTCTCTTCACAGATGCGT

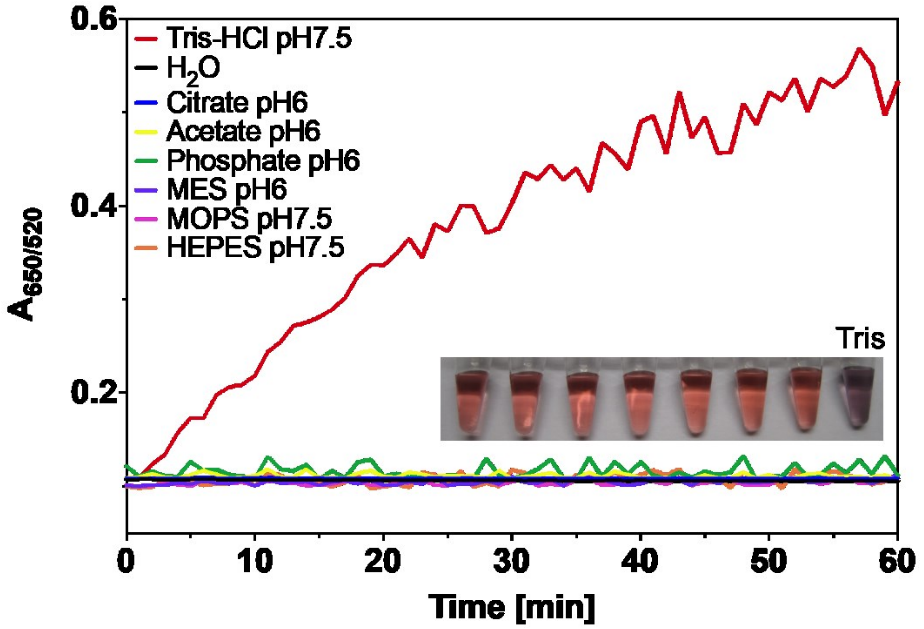

Figure S1. Kinetics of the color change of the AuNPs upon addition of the buffers (4 mM each) measured by the extinction ratio of the AuNPs at 650 nm over 520 nm. For the Tris added sample, the color gradually turned purple (inset). Tris has a primary amine that has strong affinity towards the AuNPs. After adsorption of Tris, the AuNPs were capped by non-charged hydroxyl groups leading to decreased electrostatic stabilization. Therefore, Tris cannot be used with bare AuNPs and we excluded Tris from our study.

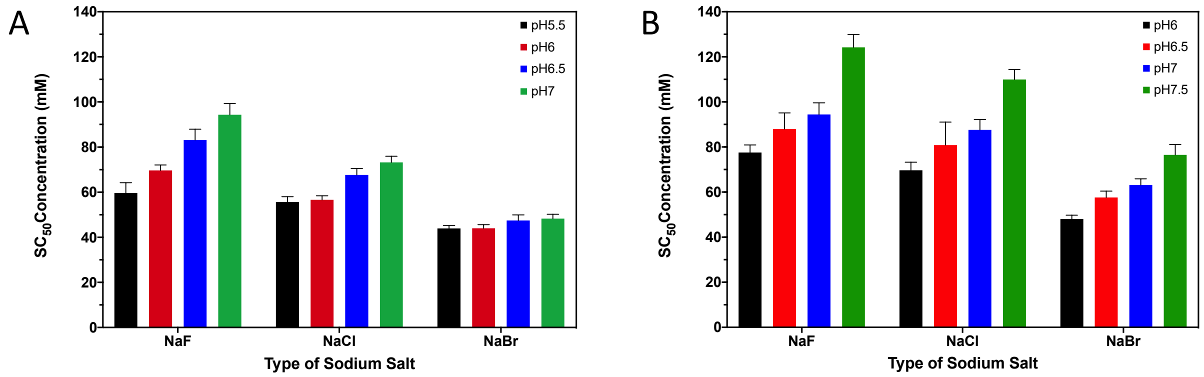

Figure S2. Salt stability of 13 nm AuNP in 4 mM (A) MES & (B) MOPS at various pH.

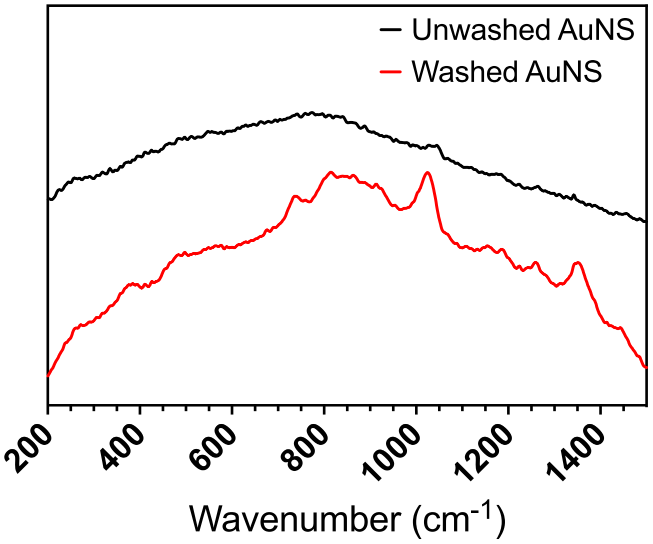

Figure S3. Raman spectra of the AuNSs before and after washing. The peaks are from the adsorbed HEPES, and washing allowed much stronger signals.
